# Aerobic and resistance exercise improves physical fitness, bone health, and quality of life in overweight and obese breast cancer survivors: a randomized controlled trial

**DOI:** 10.1186/s13058-018-1051-6

**Published:** 2018-10-19

**Authors:** Christina M Dieli-Conwright, Kerry S Courneya, Wendy Demark-Wahnefried, Nathalie Sami, Kyuwan Lee, Frank C Sweeney, Christina Stewart, Thomas A Buchanan, Darcy Spicer, Debu Tripathy, Leslie Bernstein, Joanne E Mortimer

**Affiliations:** 1 Division of Biokinesiology and Physical Therapy, University of Southern California (USC), |1540 E. Alcazar St., CHP 155, Los Angeles, CA 90089 USA; 2 Faculty of Kinesiology, Sport, and Recreation, University of Alberta, Edmonton, AB T6G 2H9 Canada; 3 Department of Nutrition Sciences, University of Alabama at Birmingham, Birmingham, AL 35294 USA; 4 Division of Endocrinology and Diabetes, Keck School of Medicine, USC, Los Angeles, CA 90033 USA; 5 Department of Medicine, Keck School of Medicine, University of Southern California, Los Angeles, CA 90033 USA; 6 Department of Breast Medical Oncology, The University of Texas MD Anderson Cancer Center, Houston, TX 77030 USA; 7 Division of Biomarkers of Early Detection and Prevention, Beckman Research Institute, City of Hope (COH), Duarte, CA 91010 USA; 8 Division of Medical Oncology and Experimental Therapeutics, COH, Duarte, CA 91010 USA

**Keywords:** Exercise, quality of life, physical fitness, bone health, breast cancer

## Abstract

**Background:**

Exercise is an effective strategy to improve quality of life and physical fitness in breast cancer survivors; however, few studies have focused on the early survivorship period, minorities, physically inactive and obese women, or tested a combined exercise program and measured bone health. Here, we report the effects of a 16-week aerobic and resistance exercise intervention on patient-reported outcomes, physical fitness, and bone health in ethnically diverse, physically inactive, overweight or obese breast cancer survivors.

**Methods:**

One hundred breast cancer survivors within 6 months of completing adjuvant treatment were assessed at baseline, post-intervention, and 3-month follow-up (exercise group only) for physical fitness, bone mineral density, serum concentrations of bone biomarkers, and quality of life. The exercise intervention consisted of moderate-vigorous (65–85% heart rate maximum) aerobic and resistance exercise thrice weekly for 16 weeks. Differences in mean changes for outcomes were evaluated using mixed-model repeated measure analysis.

**Results:**

At post-intervention, the exercise group was superior to usual care for quality of life (between group difference: 14.7, 95% CI: 18.2, 9.7; *p* < 0.001), fatigue (p < 0.001), depression (p < 0.001), estimated VO_2max_ (p < 0.001), muscular strength (p < 0.001), osteocalcin (*p* = 0.01), and BSAP (*p* = 0.001). At 3-month follow-up, all patient-reported outcomes and physical fitness variables remained significantly improved compared to baseline in the exercise group (*p* < 0.01).

**Conclusions:**

A 16-week combined aerobic and resistance exercise program designed to address metabolic syndrome in ethnically-diverse overweight or obese breast cancer survivors also significantly improved quality of life and physical fitness. Our findings further support the inclusion of supervised clinical exercise programs into breast cancer treatment and care.

**Trial registration:**

This trial is registered on ClinicalTrials.gov: NCT01140282 as of June 9, 2010.

## Background

Breast cancer survivors are at elevated risk for the development of comorbid conditions such as sarcopenia, osteoporosis, and cardiovascular disease [1] which contribute to declines in quality of life, cardiorespiratory fitness, muscular strength, and bone health. These negative health concerns are partly induced by cancer-related treatments (e.g., chemotherapy, radiation, endocrine therapy) and are exacerbated by obesity and a physically inactive lifestyle. Exercise is an effective non-pharmacologic strategy to mitigate cancer-related treatment side effects and improve quality of life, cardiorespiratory fitness, and muscular strength in breast cancer survivors [2]; however, few studies have focused on the early survivorship period (≤6 months post-treatment), minorities, physically inactive and obese women, or tested a combined exercise program and measured bone health.

The overall purpose of this trial was to compare a 16-week supervised moderate-vigorous intensity aerobic and resistance exercise intervention to usual care in physically inactive, overweight and obese breast cancer survivors. We previously reported that the exercise intervention led to significant improvements in metabolic syndrome, sarcopenic obesity, and circulating biomarkers that were maintained at 3-month follow-up [3]. Here, we report the secondary outcomes of physical fitness, bone health, and quality of life. We hypothesized that a combined exercise intervention performed within 6 months of cancer treatment completion would improve patient-reported outcomes, physical fitness, and bone health in ethnically-diverse, physically inactive, overweight/obese breast cancer survivors compared to usual care.

## Methods

### Participants/Consent

Eligible participants were < 6 months post-treatment for chemo- or radiation-therapy for stage 0-III breast cancer and were non-smokers, physically inactive (< 60 min of structured exercise/week), with BMI ≥25.0 kg/m^2^ (or body fat > 30%) and waist circumference > 88 cm. Participants were verbally screened for eligibility by phone or in person at time of consent. Treatment history and diagnosis were confirmed by medical record abstraction. Body composition measure were obtained at time of screening per testing methods described below (Covariate Measures).

Recruitment occurred between August 1, 2012 and December 31, 2016 from the USC Norris Comprehensive Cancer Center and Los Angeles County Hospital. The protocol and informed consent were IRB-approved (HS-12-00141) and registered (ClinicalTrials.gov:NCT01140282). A signed informed consent was obtained from each participant. Participants were randomized to exercise or usual care following the completion of baseline testing using concealed randomization lists.

### Experimental Design

This randomized controlled trial compared a progressive combined (aerobic and resistance) exercise intervention versus usual care on baseline to 4-month changes in physical fitness, bone health, and patient-reported outcomes. Detailed methods [4], and primary outcomes related to metabolic syndrome were published previously. Endpoints were assessed at baseline, post-intervention (month 4), and 3-month follow-up (exercise group only). To enhance participation, usual care participants were offered the exercise program following the study period.

### Cardiorespiratory Fitness

A single-stage submaximal treadmill test was used to estimate maximal oxygen uptake, VO_2max_ [5]. Participants first performed a 4-min warm up by walking on a treadmill (Desmo Woodway, Waukesha, WI) at a speed (2.0, 3.0, 4.0, or 4.5 mph) that increased their heart rate between 50 and 70% heart rate maximum. This was followed by the 4-min test at the same speed with a 5% grade; heart rate was measured during the final 30 s of the test. Using heart rate, speed, age and gender, estimated maximal oxygen uptake was predicted using the test-specific regression formula [5].

### Muscular Strength

Estimated maximal voluntary strength (1-RM) was assessed for the chest press, latissimus pulldown, knee extension, and knee flexion using the 10-repetition maximum (10-RM) method (Tuff Stuff, Pomona, CA). [6] Participants completed a warm-up load of ~ 5–8-RM before attempting 10-RM. A 2-min rest period was given between attempts; 3–5 attempts were performed.

### Dual Energy X-Ray Absorptiometry (DXA)

Dual hip and lumbar DXA scans were used to assess bone mineral density (BMD; Lunar GE iDXA, Fairfield, Connecticut).

### Blood Collection and Analysis

Fasting (≥12 h) blood was obtained by trained phlebotomists. Serum was stored at − 80° until batch analysis at study completion. Biomarkers of bone turnover were analyzed and included bone-specific alkaline phosphatase (BSAP) and osteocalcin as markers of bone formation, C-telopeptide of type 1 collagen (CTX), N-telopeptides of type 1 collagen (NTX) as markers of bone resorption, and receptor activator factor-kappa B (RANK) and receptor activator factor-kappa B ligand (RANKL) as markers of bone remodeling. In addition, we quantified calcium and 25-hydroxyvitamin D.

Osteocalcin (Meso Scale Discovery, Rockville MD, Catalog #K151HHC-1), BSAP (Ostase Assay Catalog #37300, Beckman Coulter, Ontario, Canada), CTX (Immunodiagnostics, Gaithersburg, MD, Catalog #AC-02F1), NTX (MyBioSource, San Diego, CA, Catalog #MBS705111), RANK (MyBioSource, San Diego, CA, Catalog #MBS9308775), and RANKL (MyBioSource, San Diego, CA, Catalog #MBS2533374) were analyzed by enzyme-linked immunoabsorbent assays. 25-hydroxyvitamin D was detected by high-performance liquid chromatography (Dionex Corporation, Sunnyvale, CA). Calcium was detected using an automated colorimetric technique including an ion-specific assay (abcam, Cambridge, MA Catalog #ab102505) and a colorimetric microplate reader (BioTek, Winooski, VT). Duplicate testing was performed with coefficients of variation for all samples < 10%.

### Patient-reported Outcomes

Quality of Life was assessed using the Functional Assessment of Cancer Therapy-Breast (FACT-B) and the Short Form-36 Health Survey (SF-36). The Brief Fatigue Inventory (BFI) was used to assess fatigue, where a lower score indicates less fatigue [7]. Risk for depression and depressive symptoms were assessed using the 20-item Center for Epidemiologic Studies-Depression Scale (CES-D) [8].

### Covariate Measures

Weight was measured to the nearest 0.1 kg on an electronic scale with the patient wearing a hospital gown and no shoes and height was measured to the nearest 0.5 cm with a fixed stadiometer in order to calculate BMI. Waist circumference was measured at the midpoint between the lower margin of the last palpable rib and the iliac crest. Physical activity history was assessed at baseline using an interviewer-administered, validated questionnaire to assess historical, past-year, and past-week physical activity [9]. Three-day dietary records (2 weekdays and 1 weekend day) were completed at baseline, post-intervention, and 3-month follow-up (exercise group only) within 1-week of each assessment and analyzed using Nutritionist Pro™ (Woodinville, WA). Participants completed the Charlson Comorbidity questionnaire [10]. Cancer-related information (i.e., time since treatment completion, time since diagnosis, disease stage, hormone-receptor status, endocrine therapy, and surgery) was abstracted from medical records.

### Exercise Intervention

The exercise program aligned with ACS/ACSM exercise guidelines for cancer survivors (150 min of aerobic exercise and 2–3 days of resistance exercise training/week) [11]. Participants received 3 supervised one-on-one exercise sessions/week. Days 1 and 3 consisted of aerobic and resistance exercise of ~ 80 min and Day 2 included ~ 50 min of aerobic exercise. All sessions were led by a certified ACS/ACSM Cancer Exercise Trainer. Participants wore a Polar® heart monitor (Lake Success, NY) during each exercise session. Each session began with a 5-min aerobic exercise warm-up at 40–50% estimated VO_2_max. Sequenced resistance exercise followed in circuit training fashion with no rest periods between exercises: Leg Press ⇔ Chest Press ⇒ Lunges ⇔ Seated Row ⇒ Leg Extensions ⇔ Triceps Extensions ⇒ Leg Flexion ⇔ Biceps Curl; where ⇔ indicates the two exercises that alternated until all sets were completed, then the following pair of exercises was performed. Initial resistance was set at 80% of the estimated 1-RM for lower body exercises and 60% estimated 1-RM for upper body exercises. When the participant was able to complete three sets of 10 repetitions at the set weight in two consecutive sessions then the weight is increased by 10%. Repetitions increased from 10 (week 4) to 12 (week 8) to 15 (week 12) every 4 weeks to safely build muscular endurance. Compression garments were required during the exercise sessions for all participants who held prescriptions.

Resistance exercises were followed by self-selected aerobic exercise: treadmill walking/running; rowing machine; stationary bicycle. Heart rate (HR) was monitored throughout the aerobic sessions to maintain a HR at 65–80% of maximum HR. Target HR was increased every 4 weeks to safely build cardiorespiratory endurance and to maintain the prescribed intensity as participants improved their cardiorespiratory fitness. Duration of the aerobic sessions was increased from 30 min (week 1) to 50 min (week 16) as cardiorespiratory fitness increased to meet the exercise guidelines for cancer survivors. Participants ended each session with a 5-min cool down at 40–50% estimated VO_2_max. The trainers documented attendance and minutes of exercise per session.

### Follow-up period (exercise group only)

A 12-week follow-up was instituted in the exercise group to assess intervention durability. During the 12-week period, participants were encouraged to exercise on their own without study team supervision. Participants were asked to maintain weekly physical activity logs and wear an accelerometer on a daily basis during this period; they repeated outcome measure testing upon completion of the 12-week period. Sustainability was assessed at 28-week follow-up in this group by 7-day accelerometer monitoring (Model GT3X Actigraph, Fort Walton Beach, FL). Participants were asked to wear the accelerometer during waking hours for 7 consecutive days, perform their normal or usual activity, and remove the device while bathing, showering, or swimming. Participants received verbal and written instructions and a wear time log to encourage adherence. Devices were returned at time of follow-up testing. Accelerometer data was used to estimate minutes and intensity of physical activity performed according to the manufacturer’s directions.

### Statistical Analyses

As this is a secondary analysis of the parent trial which focused on metabolic syndrome, the sample size was based on projected changes in insulin [12]. Enrollment of 100 women provided 80% statistical power (α = 0.05) to detect a 2.6 μU/ml (SD = 4.0 μU/ml) difference in mean insulin levels assuming 20% drop-out using a two-group t-test.

Within-group differences in mean changes for individual outcomes measured at post-intervention and 3-month follow-up (exercise group only) were evaluated using general linear models repeated-measures ANOVAs. Between-group differences in mean changes for individual outcomes measured at post-intervention were evaluated using mixed-model repeated measure analysis. A priori covariates included type of treatment (chemotherapy, radiation, or both), surgery type, time on hormonal therapy, comorbidities, and BMI were explored in models due to their possible associations with outcomes, but none modified results. Five women using bisphosphonates were excluded when analyzing BMD and other bone biomarkers.

Post-hoc analyses included stratification by menopausal status at time of diagnosis. (women were classified as postmenopausal if amenorrheic over the previous 12 months). Analyses were performed using SAS (Version 9.4, Cary, NC).

## Results

The study CONSORT diagram is reported elsewhere. Briefly, we assessed 418 women for eligibility of which 100 were randomized to the exercise or usual care group. Four participants in the exercise group and five participants in the usual care group did not complete the study. Baseline characteristics also are reported elsewhere and were similar across the 2 groups. On average, women were 53.5 ± 10.4 (SD) years old, postmenopausal (60%), Hispanic white (55%) or non-Hispanic white (26%), 6.2 ± 2.1 months from diagnosis, with a BMI = 33.5 ± 5.5 kg/m^2^. Women were diagnosed primarily with stage I (40%) or II (38%) breast cancer and largely treated with both chemo- and radiation- therapy (76%). The weekly average of moderate-to-vigorous physical activity at baseline was 9.6 ± 6.8 min.

Intervention adherence and adverse events are reported elsewhere. High session attendance of 96% (overall average 46 of 48 sessions) was attained by the exercise group. Adherence with aerobic exercise, and with intensity and volume of resistance exercises was 95%. No adverse events were reported over the duration of the study.

### Physical Fitness

Physical fitness outcomes are displayed in Table 1. Estimated VO_2max_, an indicator of cardiorespiratory fitness, significantly increased in the exercise group as compared to baseline and the usual care group (*p*-value< 0.001). Resting heart rate significantly decreased in the exercise group as compared to baseline and the usual care group (p-value< 0.001). Muscle strength, assessed as estimated 1-RM, significantly increased across all four exercises (leg extension, leg flexion, latissimus pulldown, chest press) in the exercise group as compared to baseline and the usual care group (*p*-values< 0.001). At follow-up, all physical fitness measures remained significantly improved in the exercise group compared to baseline (p-values< 0.001). The usual care group did not experience changes in any measure of physical fitness (p-values> 0.05).

**Table 1:** Comparison of physical fitness between exercise and usual care groups

	Baseline	Post-intervention		3-month follow-up		Between-group difference post-intervention	
	Mean (SD)	Mean (SD)	P value^a^	Mean (SD)	P value^b^	Mean (95% CI)	P value^c^
Estimated VO_2max_ (mL/kg/min)							
Exercise	23.3 (6.1)	35.1 (8.0)	< 0.001	32.1 (8.2)	< 0.001	11.8 (25.2 to 16.7)	< 0.001
Usual care	22.7 (6.4)	19.3 (8.5)	0.14	–	–		
Resting heart rate (bpm)							
Exercise	87.9 (8.0)	77.5 (8.5)	< 0.001	76.9 (8.9)	< 0.001	−15.4 (−18.1 to −11.7)	< 0.001
Usual care	87.7 (7.7)	88.1 (7.8)	0.67	–	–		
Leg extension (kgs)							
Exercise	45.4 (10.6)	75.7 (10.8)	< 0.001	77.4 (11.5)	< 0.001	30.4 (35.9 to 27.8)	< 0.001
Usual care	44.9 (9.1)	42.9 (9.0)	0.15	–	–		
Leg flexion (kgs)							
Exercise	39.5 (9.6)	63.6 (11.2)	< 0.001	62.4 (12.1)	< 0.001	24.0 (27.2 to 21.6)	< 0.001
Usual care	40.8 (10.1)	39.7 (10.6)	0.23	–	–		
Latissimus pulldown (kgs)							
Exercise	30.6 (4.7)	39.0 (6.1)	< 0.001	38.5 (6.5)	< 0.001	9.6 (11.7 to 6.1)	< 0.001
Usual care	30.3 (4.0)	29.6 (4.4)	0.27	–	–		
Chest press (kgs)							
Exercise	9.1 (2.3)	20.7 (4.5)	< 0.001	19.5 (4.0)	< 0.001	11.6 (15.9 to 7.7)	< 0.001
Usual care	9.2 (2.5)	8.7 (2.4)	0.40	–	–		

*Abbreviations*: *SD* standard deviation, *CI* confidence interval, *kgs* kilograms

^a^P value for repeated measures ANOVA comparing changes in the exercise group from baseline to post-intervention, and in the usual care group from baseline to post-intervention

^b^*P* value for repeated measures ANOVA comparing changes in the exercise group from baseline to 3-month follow-up

^c^P value for mixed model analysis comparing changes between the exercise and usual care group from baseline to post-intervention

### Bone Health

Table 2 shows baseline, post-intervention, and 3-month follow-up changes in BMD and bone biomarkers by group. Post-intervention, BMD (whole body, lumbar spine, total hip, trochanter, and femoral neck) did not significantly change in the exercise or usual care groups (*p* > 0.10). Calcium and 25-hydroxyvitamin D levels increased (*p* = 0.09) in the exercise group however this did not reach significance. Osteocalcin and BSAP, biomarkers of bone formation, increased in the exercise as compared to baseline (*p* = 0.04, 0.05, respectively) and the usual care group (*p* = 0.01, 0.07, respectively) however, significance was only reached for osteocalcin. Post-intervention, CTX and NTX, biomarkers of bone resorption, and RANK and RANKL, biomarkers of bone remodeling, did not significantly change in the exercise or usual care groups (*p* > 0.05). Stratification by menopausal status did not alter these results.

**Table 2:** Comparison of bone health between exercise and usual care groups

	Baseline	Post-intervention		3-month follow-up		Between-group difference post-intervention	
	Mean (SD)	Mean (SD)	P value^a^	Mean (SD)	P value^b^	Mean (95% CI)	P value^c^
Calcium (mg/dL)							
Exercise	10.3 (2.6)	13.7 (1.9)	0.09	13.4 (1.7)	0.10	3.4 (5.0 to 1.6)	0.10
Usual care	10.5 (2.5)	9.9 (2.6)	0.67	–	–		
25(OH)D (ng/dL)							
Exercise	21.7 (4.4)	27.8 (6.7)	0.09	27.4 (6.5)	0.09	6.1 (9.8 to 4.3)	0.10
Usual care	21.5 (4.1)	20.9 (4.0)	0.57	–	–		
Osteocalcin (ng/mL)							
Exercise	12.1 (3.1)	15.0 (4.1)	0.01	14.7 (3.5)	0.01	3.1 (5.6 to 1.5)	0.01
Usual care	12.3 (3.4)	12.0 (3.0)	0.61	–	–		
BSAP (ng/mL)							
Exercise	16.1 (4.6)	18.0 (5.0)	0.01	17.7 (4.6)	0.01	1.9 (2.4 to 0.55)	0.001
Usual care	16.2 (4.3)	15.9 (4.2)	0.55	–	–		
CTX (ng/mL)							
Exercise	0.48 (0.1)	0.44 (0.2)	0.07	0.44 (0.2)	0.07	−0.04 (−0.10 to −0.06)	0.10
Usual care	0.47 (0.1)	0.48 (0.2)	0.74	–	–		
NTX (nM BCE/L)							
Exercise	18.6 (3.1)	17.7 (2.8)	0.10	17.8 (2.7)	0.11	−0.90 (−1.1 to − 0.6)	0.12
Usual care	18.4 (2.7)	18.3 (2.5)	0.67	–	–		
RANK (pg/mL)							
Exercise	27.4 (6.8)	26.7 (6.4)	0.14	26.6 (6.1)	0.14	−0.70 (−0.9 to − 0.4)	0.20
Usual care	26.9 (6.6)	26.4 (6.5)	0.34	–	–		
RANKL (pmol/L)							
Exercise	142.5 (18.9)	146.1 (16.1)	0.09	145.7 (16.2)	0.09	3.6 (5.1 to 1.2)	0.14
Usual care	139.8 (18.1)	148.8 (18.9)	0.47	–	–		
Whole body BMD (g/cm^2^)							
Exercise	1.22 (0.1)	1.27 (0.1)	0.15	1.26 (0.1)	0.16	0.05 (0.04 to 0.02)	0.15
Usual care	1.20 (0.1)	1.19 (0.1)	0.29	–	–		
Lumbar spine BMD (g/cm^2^)							
Exercise	1.16 (0.09)	1.20 (0.09)	0.09	1.20 (0.1)	0.09	0.04 (0.03 to 0.01)	0.10
Usual care	1.15 (0.09)	1.14 (0.09)	0.57	–	–		
Total hip BMD (g/cm^2^)							
Exercise	0.91 (0.09)	0.94 (0.09)	0.17	0.94 (0.09)	0.17	0.03 (0.03 to 0.00)	0.18
Usual care	0.90 (0.09)	0.89 (0.08)	0.23	–	–		
Trochanter BMD (g/cm^2^)							
Exercise	0.72 (0.07)	0.74 (0.07)	0.18	0.74 (0.07)	0.20	0.02 (0.03 to 0.00)	0.22
Usual care	0.71 (0.06)	0.70 (0.06)	0.43	–	–		
Femoral neck BMD (g/cm^2^)							
Exercise	0.88 (0.1)	0.90 (0.1)	0.21	0.88 (0.7)	0.24	0.02 (0.03 to 0.00)	0.21
Usual care	0.87 (0.1)	0.86 (0.1)	0.23	–	–		

*Abbreviations*: *SD* standard deviation, *CI* confidence interval, *BMD* bone mineral density, *RANK* receptor activator factor-kappa B, *RANKL* receptor activator factor-kappa B ligand

^a^*P* value for repeated measures ANOVA comparing changes in the exercise group from baseline to post-intervention, and in the usual care group from baseline to post-intervention

^b^*P* value for repeated measures ANOVA comparing changes in the exercise group from baseline to 3-month follow-up

^c^P value for mixed model analysis comparing changes between the exercise and usual care group from baseline to post-intervention

### Patient-Reported Outcomes

Tables 3 and 4 display patient-reported outcomes. Post-intervention, FACT-B scores (Table 3) were significantly improved in exercise vs. usual care (between group difference: 14.7, 95% CI: 18.2, 9.7; *p* < 0.001). FACT-General, trial outcome index, and all subscales were significantly improved in the exercise group when compared to baseline (*p* ≤ 0.01) and the usual care group (p < 0.001). All SF-36 subscores (Table 4) significantly improved in the exercise group when compared to baseline and the usual care group (*p* ≤ 0.001). Fatigue and depression (Table 4) significantly reduced in the exercise group as compared to baseline (p ≤ 0.01) and the usual care group (p < 0.001). At follow-up, all patient-reported outcome measures remained significantly improved in the exercise group compared to baseline (p < 0.001).

**Table 3:** Comparison of breast cancer-specific quality of life between exercise and usual care groups

	Baseline	Post-intervention		3-month follow-up		Between-group difference post-intervention	
	Mean (SD)	Mean (SD)	P value^a^	Mean (SD)	P value^b^	Mean (95% CI)	P value^c^
Physical well-being							
Exercise	19.2 (3.1)	23.2 (3.0)	< 0.001	23.1 (3.2)	< 0.001	3.9 (7.2 to 1.7)	0.003
Usual care	19.1 (3.4)	19.1 (3.5)	0.74	–	–		
Social well-being							
Exercise	19.7 (3.0)	23.0 (3.2)	< 0.001	23.0 (3.0)	< 0.001	3.3 (6.9 to 1.1)	0.005
Usual care	19.6 (3.1)	19.5 (3.0)	0.81	–	–		
Emotional well-being							
Exercise	18.4 (2.6)	20.1 (3.1)	0.01	20.1 (3.5)	0.01	1.7 (3.4 to 0.8)	0.01
Usual care	18.3 (2.5)	18.2 (2.7)	0.75	–	–		
Functional well-being							
Exercise	19.9 (3.4)	22.0 (3.2)	0.01	21.9 (3.1)	0.01	2.0 (4.2 to 0.6)	0.01
Usual care	20.0 (3.6)	19.9 (3.3)	0.83	–	–		
Additional concerns							
Exercise	21.1 (4.7)	24.7 (4.1)	0.002	24.0 (4.5)	0.001	3.6 (7.7 to 2.0)	0.002
Usual care	21.7 (4.4)	19.6 (10.4)	0.43	–	–		
FACT-General							
Exercise	77.2 (9.0)	88.3 (9.9)	< 0.001	88.1 (9.2)	< 0.001	11.1 (16.4 to 7.7)	< 0.001
Usual care	77.0 (9.1)	76.7 (9.0)	0.54	–	–		
FACT-Breast							
Exercise	98.3 (14.1)	113.0 (13.0)	< 0.001	112.1 (13.2)	< 0.001	14.7 (18.2 to 9.7)	< 0.001
Usual care	98.7 (14.4)	96.3 (14.5)	0.44	–	–		
Trial outcome index							
Exercise	60.2 (7.3)	69.9 (7.5)	< 0.001	69.0 (7.7)	< 0.001	8.8 (15.3 to 4.3)	< 0.001
Usual care	60.8 (7.5)	58.6 (7.4)	0.64	–	–		

*Abbreviations*: *SD* standard deviation, *CI* confidence interval, *FACT* Functional Assessment of Cancer Therapy

^a^P value for repeated measures ANOVA comparing changes in the exercise group from baseline to post-intervention, and in the usual care group from baseline to post-intervention

^b^P value for repeated measures ANOVA comparing changes in the exercise group from baseline to 3-month follow-up

^c^P value for mixed model analysis comparing changes between the exercise and usual care group from baseline to post-intervention

**Table 4:** Comparison of health status, fatigue, and depression between exercise and usual care groups

	Baseline	Post-intervention		3-month follow-up		Between-group difference post-intervention	
	Mean (SD)	Mean (SD)	*P* value^a^	Mean (SD)	P value^b^	Mean (95% CI)	P value^c^
SF-36 SUBSCORES							
Physical functioning							
Exercise	65.3 (9.4)	74.1 (9.5)	0.001	73.9 (8.9)	0.001	9.2 (12.3 to 7.7)	0.001
Usual care	65.0 (9.7)	64.1 (9.8)	0.77	–	–		
Role-physical							
Exercise	68.4 (8.6)	75.7 (9.1)	0.002	74.4 (9.5)	0.003	7.3 (10.9 to 4.8)	0.001
Usual care	67.9 (9.1)	66.9 (8.7)	0.55	–	–		
Bodily pain							
Exercise	50.5 (9.6)	63.3 (11.2)	0.001	62.1 (12.1)	0.001	13.2 (17.2 to 9.6)	0.001
Usual care	50.8 (10.1)	49.7 (10.3)	0.23	–	–		
General health							
Exercise	60.1 (11.7)	67.2 (11.1)	0.002	67.0 (10.5)	0.001	7.1 (11.7 to 4.0)	0.002
Usual care	59.7 (11.0)	57.6 (10.4)	0.43	–	–		
Mental health							
Exercise	69.1 (12.3)	77.7 (12.5)	0.003	75.5 (11.0)	0.001	8.6 (12.9 to 5.7)	0.001
Usual care	69.7 (12.5)	68.7 (13.0)	0.60	–	–		
Role-emotional							
Exercise	70.1 (11.3)	83.7 (12.5)	0.001	82.0 (12.0)	0.001	12.1 (15.6 to 7.3)	0.001
Usual care	70.2 (11.5)	68.7 (10.4)	0.44	–	–		
Social functioning							
Exercise	79.1 (14.3)	87.7 (14.5)	0.001	85.5 (14.0)	0.001	8.6 (14.9 to 5.7)	0.002
Usual care	79.4 (14.5)	78.7 (12.4)	0.67	–	–		
Vitality							
Exercise	49.4 (9.3)	56.8 (9.5)	0.001	55.5 (9.0)	0.001	7.4 (12.9 to 4.3)	0.001
Usual care	49.2 (9.5)	48.7 (9.4)	0.63	–	–		
Physical component summary							
Exercise	66.1 (9.3)	72.7 (10.5)	0.003	72.0 (10.0)	0.001	6.6 (12.3 to 4.1)	0.001
Usual care	65.8 (9.0)	63.7 (9.4)	0.27	–	–		
Mental component summary							
Exercise	67.1 (11.0)	72.7 (12.1)	0.004	72.4 (12.4)	0.001	5.5 (9.6 to 3.3)	0.001
Usual care	69.2 (11.7)	68.7 (11.4)	0.56	–	–		
BFI							
Exercise	7.1 (2.0)	2.9 (1.5)	< 0.001	3.1 (1.9)	0.0001	−4.2 (−6.1 to −2.4)	< 0.001
Usual care	7.2 (2.1)	7.7 (2.4)	0.30	–	–		
CES-D							
Exercise	15.1 (3.3)	9.7 (2.5)	< 0.001	9.5 (3.0)	0.0001	6.6 (11.9 to 3.1)	< 0.001
Usual care	15.2 (3.5)	16.7 (3.4)	0.11	–	–		

*Abbreviations*: *SD* standard deviation, *CI* confidence interval; Short form-36 Health Status, *SF-36*; Brief Fatigue Index, *BFI*; Center for Epidemiological Studies Depression; *CES-D*

^a^P value for repeated measures ANOVA comparing changes in the exercise group from baseline to post-intervention, and in the usual care group from baseline to post-intervention

^b^*P* value for repeated measures ANOVA comparing changes in the exercise group from baseline to 3-month follow-up

^c^P value for mixed model analysis comparing changes between the exercise and usual care group from baseline to post-intervention

## Discussion

A supervised 16-week aerobic and resistance exercise intervention designed to improve metabolic syndrome also led to significant improvements in quality of life, depression, fatigue, and physical fitness that were maintained at 3-month follow-up among ethnically-diverse, physically inactive, and overweight/obese breast cancer survivors. While the intervention did not alter bone density, osteocalcin and BSAP showed significant improvements. This is the first study to our knowledge to significantly improve these outcomes with a structured combined exercise intervention in an ethnically-diverse sample of overweight or obese breast cancer survivors soon after treatment. These results are impactful given that quality of life, fatigue, and physical deconditioning are some of the most common and persevering symptoms reported by breast cancer survivors [13, 14]. This work supports the ACS/ACSM exercise guidelines for cancer survivors and demonstrates successful integration of these guidelines for women from different ethnic backgrounds.

Remarkable improvements in patient-reported outcomes were observed for quality of life, fatigue, and depressive symptoms. While our results align with those reported in the literature [15, 16], the exercise-induced reductions in fatigue (effect size *d* = 0.91) and depressive symptomology (effect size *d* = 0.97) are unparalleled with substantially larger effect sizes than the 0.30 and 0.38 reported from recent meta-analyses examining exercise and fatigue [16] and depressive symptoms [17], respectively, in cancer survivors. The marked reduction in these two domains may be due to the inclusion of women within a short period (6 months) of concluding cancer-related treatment, the physically inactive and obese nature of the participants upon enrollment, and the ethnically diverse sample. Further, it is plausible that the combination of aerobic and resistance exercise, in a supervised manner, resulted in greater benefits on patient-reported outcomes than only one mode of exercise. Previous studies have integrated a supervised combined exercise intervention with significant improvements in quality of life [18–23], fatigue [20], and depression [18] yet achieved a magnitude of improvement lower than our results. Differences in exercise duration, intensity, and frequency may underlie the various magnitudes of change across outcomes.

Physical function, described as the ability of an individual to perform common daily activities has been shown to predict survival and mortality in breast cancer survivors [24, 25], and thus is gaining support as a relevant prognostic indicator among cancer survivors. One determinant of physical function is one’s level of physical fitness, thereby improving physical fitness as a means to improve physical function is of high importance. We found notable improvements in estimated VO_2max_ (52%) and muscular strength (> 30%) following exercise. For example, estimated 1-RM for chest press increased by 133%. Previous studies utilizing a supervised combined exercise intervention have produced significant improvements in physical fitness [18–23, 26] yet to a lesser degree than our results. Various exercise fitness testing methodologies (i.e., six-minute walk test, 12-min walk test, Aerobic Power Index cycle test, modified Bruce treadmill test) were used across studies, challenging the interpretation of results and between-study comparisons. Moreover, we used indirect assessment of VO_2max_ that involved the use of a regressions formula which may have influenced our results.

Our large exercise-induced effects on physical fitness may be due to the high rate of physically inactive behavior at baseline, and subsequent low physical fitness levels at baseline, the early survivorship phase, high adherence, a supervised environment, and inclusion of both aerobic and resistance exercise. In particular, most (95%) of our Hispanic white participants had no history of physical activity and therefore may have experienced greater gains in the outcomes from the intervention. The large increase in maximal strength for the chest press may be attributed to the deconditioned status of the participants enrolled within 6 months of the completion of cancer treatment. Our adherence of 96% exceeds the 70–80% noted in other trials [27–29], and could be attributed to flexible session timing (5 am – 8 pm, 7d/wk), one-on-one supervision, and the provision of parking permits or bus passes to overcome transportation barriers. Intentionally, we conducted the intervention in a controlled clinical setting under direct supervision to ensure exercise safety and dose intensity needed to elicit greater benefits in our outcomes.

Bone loss occurs as a consequence of breast cancer treatment [30]. Premenopausal breast cancer survivors may experience chemotherapy-induced amenorrhea or pharmacologic ovarian suppression treatment, predisposing them to further bone loss [30]. While we did not observe improvements in BMD, this may be explained by the short duration of our intervention. Interventions similar to ours that included aerobic and resistance exercise produced conflicting results and involved varying durations of exposure to exercise. Thomas et al. did not observe significant improvements in BMD following a 12-month aerobic and resistance exercise intervention in breast cancer survivors taking aromatase inhibitors (change from baseline: 0.001, 95% CI: -0.009, 0.010) [31]. Almstedt and Tarleton [32] observed improvements in T-scores at the femoral neck and whole body following 13 weeks of aerobic and resistance exercise + whole body vibration in female cancer survivors (breast cancer *n* = 5). It is probable that the incorporation of whole body vibration contributed to the improvements BMD observed by Almstedt and Tarleton. Notably, we did observe a significant increase in osteocalcin and BSAP, a biomarkers of bone formation, so perhaps a longer intervention of a minimum of 6 months would elicit a positive effect on BMD.

Strengths of our study include the focus on high-risk breast cancer survivors with high rates of inactivity and obesity, targeting the early survivorship period, the ethnically-diverse sample, the randomized controlled trial design, the high adherence rate, and the modest loss-to-follow up. Limitations include lack of direct physical function and physical fitness measures (i.e., 1-RM and VO2max), and lack of an attention control group.

## Conclusions

In summary, a combined exercise intervention designed to improve metabolic syndrome in ethnically diverse, overweight or obese BCS also demonstrated significant improvements in patient-reported outcomes and physical fitness. Based on our findings, supervised clinical exercise programs that adhere to the ACS/ACSM exercise guidelines should be incorporated into breast cancer treatment and early survivorship care plans.

## Abbreviations

ACS: American Cancer Society
ACSM: American College of Sports Medicine
ANOVA: analysis of variance
BFI: Brief Fatigue Inventory
BMD: bone mineral density
BMI: body mass index
BSAP: bone-specific alkaline phosphatase
CES-D: Center for Epidemiologic Studies-Depression Scale
CTX: C-telopeptide of type 1 collagen
DXA: dual energy x-ray absorptiometry
FACT-B: Functional Assessment of Cancer Therapy-Breast
HR: heart rate
IRB: institutional review board
NTX: N-telopeptides of type 1 collagen
RANK: Receptor activator factor-kappa B
RANKL: Receptor activator factor-kappa B ligand
RM: repetition maximum
SD: standard deviation
SF-36: Short Form-36 Health Survey
